# FLOT versus SOX as immunochemotherapy backbones for neoadjuvant or conversion therapy in gastric cancer: efficacy, toxicity, and postoperative safety in a real-world cohort

**DOI:** 10.3389/fimmu.2026.1890256

**Published:** 2026-09-29

**Authors:** Penghui Liu, Na Li, Jiwu Guo, Jizhen Wang, Ziyuan Mou, Jie Mao

**Affiliations:** Department of General Surgery, Lanzhou University Second Hospital, Lanzhou, China

**Keywords:** conversion therapy, FLOT, gastric cancer, immune checkpoint inhibitor, neoadjuvant therapy, SOX

## Abstract

**Objective:**

To compare FLOT and SOX as chemotherapy backbones for neoadjuvant or conversion immunochemotherapy in gastric cancer, with emphasis on pathological efficacy, treatment-related toxicity, treatment feasibility, postoperative safety, and exploratory survival outcomes.

**Methods:**

This retrospective real-world cohort study included 148 patients with gastric cancer who received preoperative immune checkpoint inhibitor-based therapy, with or without targeted therapy, followed by gastrectomy with D2 lymphadenectomy from 2021 to 2025. Patients were grouped according to chemotherapy backbone: FLOT-based immunochemotherapy (n = 75) or SOX-based immunochemotherapy (n = 73). The primary endpoint was grade 3 or higher treatment-related adverse events. Secondary endpoints included pathological complete response, major pathological response, ypN0 status, R0 resection, adverse event-related treatment modification, postoperative complications, disease-free survival, and overall survival. Exploratory survival analyses included Kaplan–Meier estimation and Firth-penalized Cox proportional hazards regression to account for the limited number of survival events. All subgroup analyses were exploratory and hypothesis-generating.

**Results:**

FLOT and SOX achieved comparable pathological efficacy. Major pathological response occurred in 42.7% of patients in the FLOT group and 45.2% in the SOX group, while pathological complete response occurred in 18.7% and 27.4%, respectively. ypN0 status, clinical-to-pathological downstaging, and R0 resection rates were also similar. Grade 3 or higher adverse events were numerically more frequent with FLOT than SOX (46.7% vs. 32.9%), as were adverse event-related dose reduction or treatment interruption (36.0% vs. 21.9%). However, postoperative complications were comparable between groups (21.3% vs. 23.3%). Sensitivity analyses excluding targeted therapy showed consistent findings. Exploratory survival analyses were limited by 32 DFS events and 16 deaths and by markedly shorter follow-up in the SOX group. Although the unadjusted hazard ratios numerically favored FLOT for both DFS and OS, multivariable Firth-penalized Cox models substantially attenuated these estimates (DFS adjusted HR, 1.08; 95% CI, 0.50–2.34; OS adjusted HR, 1.56; 95% CI, 0.53–4.60). Accordingly, no definitive conclusions regarding survival superiority, inferiority, or equivalence could be drawn.

**Conclusion:**

In this cohort, FLOT and SOX showed broadly comparable pathological efficacy and postoperative complication rates, whereas FLOT was associated with a numerically greater preoperative toxicity and treatment-modification burden. SOX may therefore represent a balanced option in selected patients, but these findings require prospective validation.

## 1 Introduction

Gastric cancer remains a major global health burden and continues to rank among the leading causes of cancer-related mortality worldwide (1). For patients with locally advanced gastric or gastroesophageal junction adenocarcinoma, multimodal treatment integrating systemic therapy and radical surgery has become an important strategy to improve tumor control and long-term outcomes (2). In Western practice, perioperative chemotherapy with fluorouracil, leucovorin, oxaliplatin, and docetaxel, known as the FLOT regimen, has been established as a standard treatment backbone after the FLOT4 trial demonstrated superior survival compared with epirubicin-based triplet chemotherapy (3).

However, treatment strategies for gastric cancer differ substantially between Western and East Asian practice. In East Asia, where D2 lymphadenectomy is routinely performed by experienced surgical teams, fluoropyrimidine–platinum doublets remain widely used in perioperative and adjuvant settings (4, 5). Among these regimens, S-1 plus oxaliplatin (SOX) is highly relevant to Asian practice because of its feasibility and compatibility with D2-based perioperative treatment pathways (4–6). The RESOLVE trial further supported the role of SOX-based perioperative or postoperative therapy in patients with locally advanced gastric or gastroesophageal junction adenocarcinoma undergoing D2 gastrectomy (4, 5). This regional divergence raises an increasingly important clinical question: whether treatment intensification with FLOT provides additional benefit over SOX in Asian patients, particularly when immune checkpoint inhibitors are incorporated into preoperative treatment.

Immune checkpoint inhibitors have rapidly moved from the metastatic setting into perioperative and neoadjuvant treatment for gastric cancer. In the final analysis of KEYNOTE-585, perioperative pembrolizumab plus chemotherapy improved pathological complete response and yielded an event-free survival hazard ratio of 0.81 and an overall survival hazard ratio of 0.86, although the survival endpoints did not meet the prespecified statistical criteria for significance (7). In DANTE, atezolizumab combined with perioperative FLOT improved pathological regression and downstaging signals in resectable esophagogastric adenocarcinoma (8). More recently, the phase III MATTERHORN trial demonstrated a significant event-free survival benefit with perioperative durvalumab plus FLOT (HR, 0.71), with 24-month event-free survival rates of 67% versus 59% with FLOT alone (9). Nevertheless, these trials primarily addressed whether immunotherapy should be added to chemotherapy, whereas the optimal chemotherapy backbone for immunotherapy remains less clearly defined.

The choice of chemotherapy backbone is clinically important because efficacy must be balanced against treatment-related toxicity, treatment completion, surgical timing, and postoperative recovery. FLOT is a more intensive triplet regimen and may theoretically enhance tumor regression through greater cytotoxic activity, but this potential benefit may come at the cost of higher hematologic and non-hematologic toxicity, dose reduction, treatment interruption, or delayed surgery. By contrast, SOX is a widely used doublet regimen in Asian practice and may offer a more tolerable and practical backbone for combination with immunotherapy. Previous head-to-head evidence comparing neoadjuvant FLOT and SOX before the immunotherapy era did not demonstrate a clear difference in pathological response or postoperative morbidity (10). More recent studies have expanded the comparative evidence base. Li et al. reported broadly comparable pathological response, survival, and safety outcomes with PD-1 inhibitor plus FLOT versus PD-1 inhibitor plus SOX in a retrospective cohort (11), while Sah et al. reported comparable long-term overall and disease-free survival between neoadjuvant FLOT and SOX in the updated secondary analysis of a randomized phase II trial (12). Nevertheless, evidence remains limited for broader real-world populations receiving neoadjuvant or conversion immunochemotherapy, particularly in settings involving heterogeneous immunotherapy backgrounds, targeted therapy, treatment feasibility, and postoperative safety.

Therefore, the relevant clinical question in contemporary Asian practice is not simply whether FLOT is more active than SOX, but whether any incremental pathological benefit, if present, is sufficient to justify greater treatment burden. This issue is particularly important in real-world settings, where regimen selection is influenced by disease extent, performance status, organ function, treatment goals, biomarker information, drug accessibility, and multidisciplinary assessment. Whether higher preoperative treatment toxicity translates into increased postoperative morbidity after D2 gastrectomy also remains uncertain.

To address this gap, we conducted a real-world cohort study of patients with gastric cancer who received neoadjuvant or conversion immunochemotherapy followed by gastrectomy with D2 lymphadenectomy. We compared FLOT and SOX as chemotherapy backbones in combination with immunotherapy, with or without targeted therapy, focusing on treatment-related toxicity, pathological response, treatment feasibility, postoperative safety, and exploratory survival outcomes. We hypothesized that FLOT and SOX may achieve comparable pathological efficacy in the immunotherapy setting, whereas FLOT may be associated with greater severe toxicity and treatment modification risk without necessarily increasing postoperative complications.

## 2 Materials and methods

### 2.1 Study design and patients

This retrospective real-world cohort study was conducted at the Second Hospital of Lanzhou University. Patients were identified from an institutional gastric cancer database of consecutive patients who received preoperative systemic therapy followed by gastrectomy from 2021 to 2025. For the present analysis, we focused on patients who received immune checkpoint inhibitor-based preoperative treatment, with or without targeted therapy.

The inclusion criteria were as follows: (1) histologically confirmed gastric adenocarcinoma; (2) receipt of neoadjuvant or conversion immunochemotherapy before surgery; (3) use of either FLOT or SOX as the chemotherapy backbone; (4) subsequent gastrectomy with D2 lymphadenectomy; and (5) available clinicopathological, treatment-related adverse event, postoperative safety, and follow-up data. The exclusion criteria were as follows: (1) receipt of chemotherapy alone without immunotherapy; (2) use of chemotherapy backbones other than FLOT or SOX; (3) absence of gastrectomy with D2 lymphadenectomy after preoperative treatment; and (4) incomplete key clinicopathological, safety, or follow-up data. Patients receiving chemotherapy alone were excluded from the main analysis to avoid overlap with previous treatment-pattern comparisons and to specifically evaluate FLOT versus SOX as immunochemotherapy backbones.

Neoadjuvant therapy was generally administered to patients with clinically locally advanced, potentially resectable disease. Conversion therapy was considered for selected patients with initially unresectable or limited metastatic disease. After systemic therapy, resectability was reassessed by the multidisciplinary team based on disease control on post-treatment imaging, endoscopic information when available, performance status, organ function, and technical surgical feasibility. Surgery was undertaken only when the disease was considered technically resectable and the patient was deemed fit for gastrectomy. Clinical staging was determined using pretreatment imaging, endoscopic findings, and pathological confirmation when available, according to the eighth edition of the American Joint Committee on Cancer staging system (13).

A total of 148 patients who received preoperative immunochemotherapy were included in the final analysis, including 75 patients in the FLOT group and 73 patients in the SOX group. All patients underwent gastrectomy with D2 lymphadenectomy.

### 2.2 Treatment regimens and grouping

Patients were grouped according to the chemotherapy backbone used during preoperative immunochemotherapy. The FLOT group included patients who received fluorouracil, leucovorin, oxaliplatin, and docetaxel in combination with an immune checkpoint inhibitor, with or without targeted therapy. The SOX group included patients who received S-1 plus oxaliplatin in combination with an immune checkpoint inhibitor, with or without targeted therapy.

Immune checkpoint inhibitors included PD-1-based agents and PD-1/CTLA-4 bispecific blockade. Targeted therapy included anti-HER2-directed agents (trastuzumab and disitamab vedotin) and anti-angiogenic agents (apatinib and ramucirumab). These agents were administered in selected patients according to biomarker status, disease characteristics, treatment goals, drug availability, and multidisciplinary assessment.

The choice of FLOT or SOX was not randomized. In routine multidisciplinary decision-making, overall fitness and expected treatment tolerance, including ECOG performance status, comorbidities, and organ function, together with disease extent and tumor burden, were the principal clinical considerations. Age, biomarker status, patient preference, and drug accessibility were considered within this broader clinical context rather than used as isolated decision rules. Because the individual rationale for chemotherapy-backbone selection was not prospectively coded, the relative contribution of each factor could not be quantified.

### 2.3 Surgical assessment and procedure

Surgical resectability and operative timing were assessed before surgery by the multidisciplinary team based on pretreatment disease extent, post-treatment imaging findings, endoscopic information, performance status, organ function, and surgical feasibility. Surgery was performed when the tumor was considered technically resectable and the patient was deemed fit for gastrectomy. The type of gastrectomy was selected according to tumor location, disease extent, and surgical judgment. D2 lymphadenectomy was performed as the standard surgical procedure for all included patients, in accordance with gastric cancer surgical principles in East Asian practice (14).

### 2.4 Pathological evaluation

Resected specimens were evaluated by experienced gastrointestinal pathologists according to institutional protocols. Pathological staging was recorded using ypT and ypN categories. Pathological tumor regression was assessed using the Becker tumor regression grading system (15).

Pathological complete response was defined as the absence of residual viable tumor in both the primary tumor and regional lymph nodes, corresponding to ypT0N0. Major pathological response was defined as 10% or less residual viable tumor in the primary lesion, including pathological complete response. ypN0 was defined as the absence of metastatic lymph nodes in the resected specimen. Clinical-to-pathological downstaging was defined as a reduction in overall stage from pretreatment clinical TNM stage to postoperative ypTNM stage. R0 resection was defined as microscopically margin-negative resection.

### 2.5 Treatment-related safety assessment

Treatment-related adverse events were collected from medical records during preoperative systemic therapy. Adverse events were graded according to the National Cancer Institute Common Terminology Criteria for Adverse Events version 5.0 (16). The primary endpoint of this study was the occurrence of grade 3 or higher treatment-related adverse events.

Secondary treatment-related safety and feasibility endpoints included any-grade adverse events, serious adverse events, immune-related adverse events, adverse event-related dose reduction or treatment interruption, adverse event-related permanent discontinuation, number of treatment cycles, and treatment-to-surgery interval. The treatment-to-surgery interval was defined as the time from initiation of preoperative systemic therapy to the date of surgery. Serious adverse events were defined as adverse events resulting in hospitalization or prolonged hospitalization, life-threatening events, significant disability, or other clinically serious outcomes documented in medical records. Immune-related adverse events were identified retrospectively based on the treating physician’s documented attribution of an immune-mediated etiology, considering the temporal relationship with immunotherapy, organ-specific clinical manifestations, laboratory or imaging findings, treatment records, and competing alternative causes. Events without documented immune-mediated attribution were classified as conventional treatment-related adverse events rather than irAEs. No independent central adjudication of irAEs was performed.

### 2.6 Postoperative outcomes

Postoperative outcomes were collected from surgical and inpatient records. The main postoperative safety endpoint was the occurrence of any postoperative complication. Specific complications included anastomotic leakage, pulmonary infection, wound infection, abdominal effusion, pleural effusion, postoperative bleeding, abdominal distension, and other recorded complications. Postoperative hospital stay was defined as the number of days from surgery to discharge. Postoperative complications were evaluated during the index hospitalization and within 30 days after surgery.

### 2.7 Follow-up and survival outcomes

Patients were followed through outpatient visits, inpatient records, imaging examinations, telephone follow-up, and institutional medical records. Disease-free survival (DFS) was defined as the time from the date of surgery to disease recurrence, disease progression, or death from any cause, whichever occurred first. Patients without a DFS event were censored at the date of the last available disease assessment. Overall survival (OS) was defined as the time from the date of surgery to death from any cause, with patients who remained alive censored at the date they were last known to be alive. Follow-up duration was summarized separately for DFS and OS as median with interquartile range. Survival outcomes were considered exploratory because of the retrospective design, limited number of events, and heterogeneity in follow-up duration. The study was not designed or statistically powered to detect between-group differences in DFS or OS.

### 2.8 Study endpoints

The primary endpoint was grade 3 or higher treatment-related adverse events. Key secondary endpoints included pathological efficacy, treatment feasibility, postoperative safety, and exploratory survival outcomes. Pathological efficacy endpoints included pathological complete response, major pathological response, ypN0, Becker tumor regression grade, clinical-to-pathological downstaging, and R0 resection. Treatment feasibility endpoints included serious adverse events, immune-related adverse events, adverse event-related dose reduction or treatment interruption, adverse event-related permanent discontinuation, treatment cycles, and treatment-to-surgery interval. Postoperative safety endpoints included overall and specific postoperative complications and postoperative hospital stay. Disease-free survival and overall survival were analyzed as exploratory endpoints.

### 2.9 Statistical analysis

Continuous variables were summarized as mean with standard deviation or median with interquartile range, depending on distribution. Categorical variables were summarized as frequencies and percentages. Between-group comparisons were performed using the Student’s t test or Mann–Whitney U test for continuous variables, and the χ² test or Fisher’s exact test for categorical variables, as appropriate. Baseline balance between treatment groups was additionally assessed using absolute standardized mean differences; generalized standardized mean differences were used for categorical variables with more than two levels. The 95% confidence intervals for proportions displayed in **Figure 1** were calculated using the Wilson method. No *a priori* sample size calculation was performed because this retrospective study included all eligible patients treated during the study period. To contextualize the statistical precision of the primary endpoint, a *post hoc* power analysis was performed for grade 3 or higher treatment-related adverse events using the observed event rates in the FLOT and SOX groups, a two-sided α of 0.05, and the actual group sizes. This analysis was considered descriptive and was not used to redefine statistical significance.

**Figure 1:**
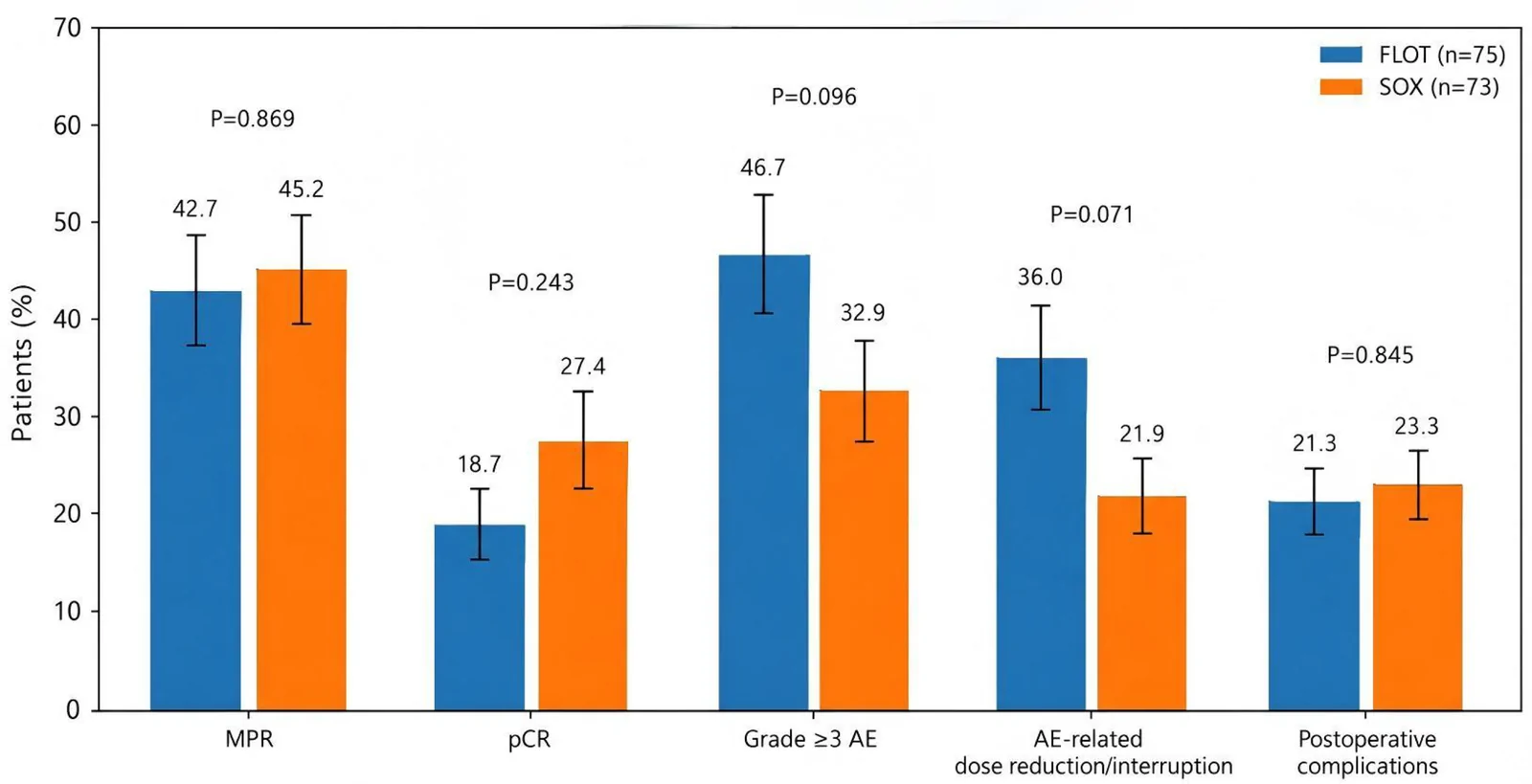
Key efficacy, toxicity, and safety outcomes. Comparison of major pathological response, pathological complete response, grade 3 or higher adverse events, adverse event-related dose reduction or treatment interruption, and postoperative complications between the FLOT and SOX groups. Error bars represent 95% confidence intervals for binomial proportions calculated using the Wilson method. AE, adverse event; FLOT, fluorouracil, leucovorin, oxaliplatin, and docetaxel; MPR, major pathological response; pCR, pathological complete response; SOX, S-1 plus oxaliplatin. MPR included patients with pCR.

The association between chemotherapy backbone and key binary outcomes was evaluated using logistic regression models, with SOX as the reference group. Odds ratios and 95% confidence intervals were calculated. Three prespecified multivariable logistic regression models were constructed for grade 3 or higher treatment-related adverse events, major pathological response, and postoperative complications. Covariates were selected based on clinical relevance, baseline imbalance, and the number of outcome events to reduce the risk of model overfitting. For each model, the number of covariates was limited according to the number of outcome events. Candidate covariates included baseline demographic and disease-related factors, such as age, sex, body mass index, ECOG performance status, clinical T stage, clinical N stage, clinical M status, PD-L1 combined positive score status, immunotherapy agent, and targeted therapy. For the prespecified logistic regression models, treatment exposure variables, including number of treatment cycles and treatment-to-surgery interval, were interpreted descriptively and were not included in the primary adjusted models to avoid potential overadjustment.

Sensitivity analyses were performed to assess the robustness of the main findings. First, patients receiving targeted therapy were excluded to generate an immunochemotherapy-only cohort. Second, analyses were repeated after excluding patients with initially unresectable or metastatic disease when appropriate. Additional sensitivity analyses were performed to address the imbalance in immunotherapy background. First, treatment effects on grade 3 or higher adverse events, MPR, pCR, and postoperative complications were estimated using Mantel–Haenszel analyses stratified by individual immune checkpoint inhibitor agent. Only ICI strata containing both FLOT- and SOX-treated patients contributed to the common odds-ratio estimates. Second, an inverse probability of treatment weighting (IPTW) analysis was performed in the common-support population restricted to ICI agents represented in both chemotherapy-backbone groups. Propensity scores for receipt of FLOT versus SOX were estimated using logistic regression including age, sex, BMI, ECOG performance status, pretreatment cT, cN, and cM status, PD-L1 CPS status, targeted therapy use, and individual ICI agent. Stabilized weights were truncated at the 2.5th and 97.5th percentiles to limit the influence of extreme weights. Covariate balance was assessed using standardized mean differences, with an absolute standardized mean difference <0.10 considered acceptable. Weighted logistic regression with robust standard errors was used to estimate IPTW-adjusted odds ratios and 95% confidence intervals. Third, exploratory subgroup analyses were conducted according to age (<60 vs. ≥60 years), targeted therapy use, PD-L1 CPS status (<5 vs. ≥5), and immunotherapy background. All subgroup analyses were considered hypothesis-generating. For the primary endpoint of grade 3 or higher treatment-related adverse events, potential treatment-effect heterogeneity was formally assessed using treatment-by-subgroup interaction terms in logistic regression models. Interaction P values were calculated for age, targeted therapy use, PD-L1 CPS status, and immunotherapy background. For interaction testing, immunotherapy background was categorized as cadonilimab versus single-agent PD-1 blockade because the numbers receiving other individual PD-1 inhibitors were small. No adjustment for multiple comparisons was performed; therefore, interaction P values were interpreted descriptively and were not considered confirmatory evidence of treatment-effect heterogeneity. Subgroup analyses of secondary efficacy and safety outcomes were summarized descriptively without formal effect-modification inference.

Kaplan–Meier methods were used to estimate DFS and OS, with between-group comparisons performed using the log-rank test. Median follow-up duration with interquartile range was summarized separately for DFS and OS in each treatment group. Twelve-month DFS and OS probabilities with 95% confidence intervals were additionally estimated as fixed-time descriptive summaries. Numbers at risk were displayed beneath the Kaplan–Meier curves.

Cox proportional hazards regression was used to estimate hazard ratios (HRs) and 95% confidence intervals for FLOT versus SOX. Because only 32 DFS events and 16 deaths were observed and the distribution of survival events was sparse across treatment backgrounds, multivariable survival analyses were performed using Firth-penalized Cox regression to reduce small-sample bias and model instability. The baseline-adjusted model included age, ECOG performance status, pretreatment cT, cN, and cM components, PD-L1 CPS status, immunotherapy background, and targeted therapy. Age was modeled per 10-year increase; ECOG performance status, cT, and cN were modeled ordinally per one-category increase; cM was categorized as cM1 versus cM0; PD-L1 CPS was categorized as ≥5 versus <5; and immunotherapy background was categorized as cadonilimab versus single-agent PD-1 blockade. Because the available dataset did not contain a separate pretreatment clinical stage-group variable, clinical disease extent was represented by the cT, cN, and cM components. An extended adjusted model additionally included treatment-to-surgery interval as a continuous variable per 1-week increase. Because treatment-to-surgery interval occurs after treatment assignment and may reflect treatment toxicity, response assessment, and treatment scheduling, the baseline-adjusted and extended models are reported separately.

The proportional hazards assumption for the chemotherapy-backbone term was assessed using Schoenfeld residual-based diagnostics. Exploratory subgroup survival analyses were performed according to age, targeted therapy use, PD-L1 CPS status, immunotherapy background, and clinical metastatic status. Subgroup HRs were derived from unadjusted Cox models and presented with 95% confidence intervals. No adjustment for multiple comparisons was performed, and all subgroup survival analyses were considered descriptive and hypothesis-generating.

All statistical tests were two-sided, and P values less than 0.05 were considered statistically significant. Given the retrospective and exploratory nature of subgroup and survival analyses, findings were interpreted cautiously, with emphasis on effect sizes, confidence intervals, and clinical consistency rather than statistical significance alone. Statistical analyses were performed using SPSS version 26.0 (IBM Corp., Armonk, NY, USA) and R version 4.3.2.

## 3 Results

### 3.1 Patient characteristics and treatment exposure

A total of 148 patients who received preoperative immunochemotherapy followed by gastrectomy with D2 lymphadenectomy were included in the final analysis. Among them, 75 patients received FLOT-based immunochemotherapy and 73 received SOX-based immunochemotherapy. The study flowchart is shown in **Figure 2**.

**Figure 2:**
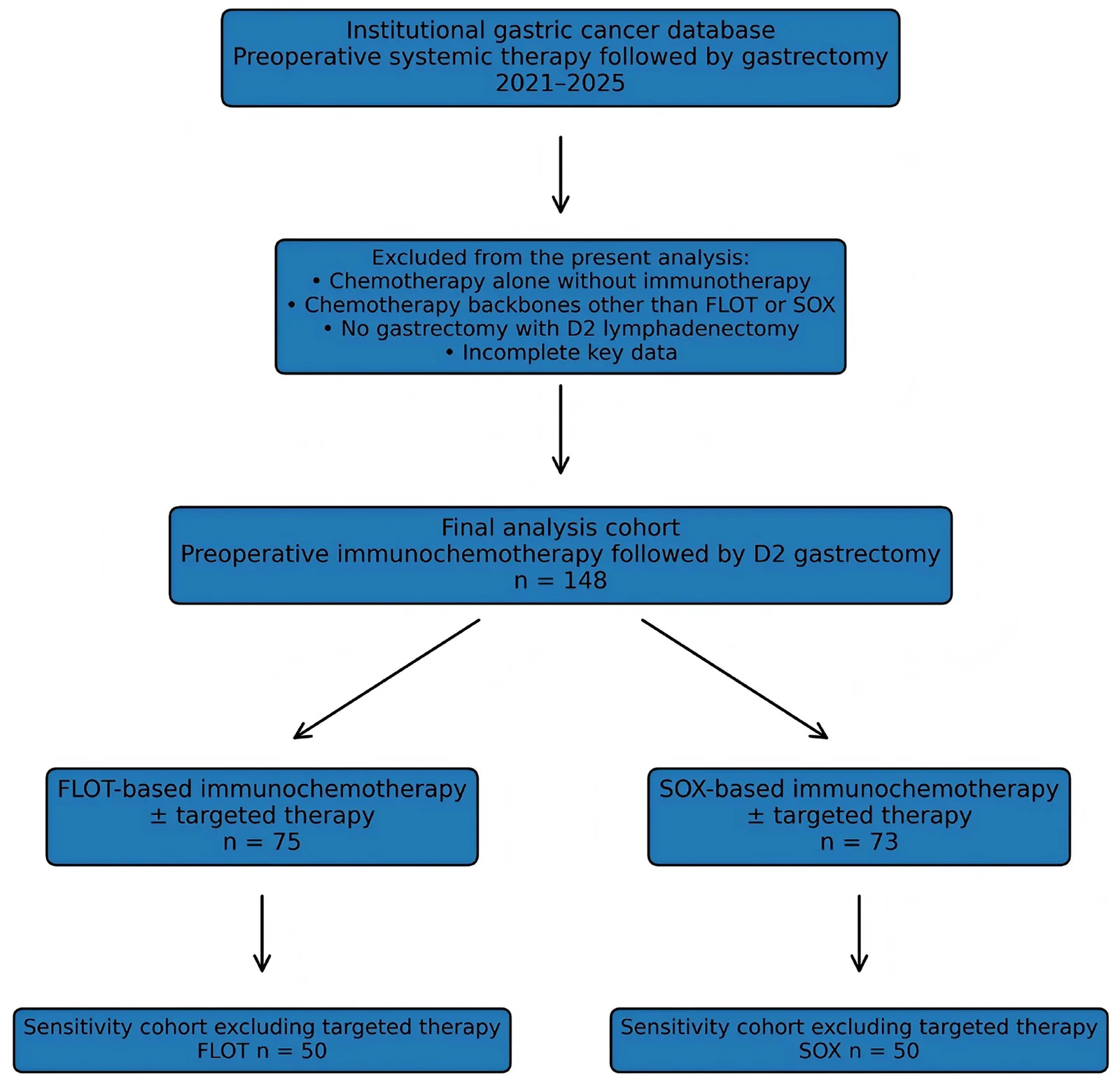
Patient selection and study flowchart. A total of 148 patients receiving preoperative immunochemotherapy followed by D2 gastrectomy were included, comprising 75 patients in the FLOT group and 73 patients in the SOX group. Sensitivity analyses excluding targeted therapy included 50 patients in each group. Abbreviations: FLOT, fluorouracil, leucovorin, oxaliplatin, and docetaxel; SOX, S-1 plus oxaliplatin.

Baseline demographic and clinicopathological characteristics were generally similar between the two groups (**Table 1**), although standardized mean differences indicated varying degrees of imbalance across several characteristics. More pronounced imbalance was observed in treatment background, particularly in the distribution of immune checkpoint inhibitors and targeted therapy classes.

**Table 1:** Baseline characteristics and treatment exposure.

Variable	FLOT (n=75)	SOX (n=73)	Absolute SMD
Baseline characteristics			
Age, years	58.0 (52.0–66.5)	60.0 (55.0–66.0)	0.20
Sex			0.03
Male	64/75 (85.3)	63/73 (86.3)	
Female	11/75 (14.7)	10/73 (13.7)	
BMI, kg/m²	23.0 (22.3–23.6)	22.9 (22.4–23.7)	0.10
ECOG performance status			0.36
0	10/75 (13.3)	3/73 (4.1)	
1	61/75 (81.3)	63/73 (86.3)	
2	4/75 (5.3)	7/73 (9.6)	
Tumor location			0.21
Cardia	29/75 (38.7)	27/73 (37.0)	
Body	18/75 (24.0)	24/73 (32.9)	
Antrum	28/75 (37.3)	22/73 (30.1)	
Lauren classification			0.16
Intestinal	39/75 (52.0)	42/73 (57.5)	
Diffuse	27/75 (36.0)	21/73 (28.8)	
Mixed	9/75 (12.0)	10/73 (13.7)	
Differentiation			0.38
Moderately differentiated	5/75 (6.7)	0/73 (0.0)	
Moderate-to-poorly differentiated	42/75 (56.0)	42/73 (57.5)	
Poorly differentiated	28/75 (37.3)	31/73 (42.5)	
Borrmann type			0.25
II	8/75 (10.7)	3/73 (4.1)	
III	61/75 (81.3)	64/73 (87.7)	
IV	6/75 (8.0)	6/73 (8.2)	
Clinical T stage			0.24
cT2	4/75 (5.3)	2/73 (2.7)	
cT3	42/75 (56.0)	35/73 (47.9)	
cT4	29/75 (38.7)	36/73 (49.3)	
Clinical N stage			0.28
cN0	7/75 (9.3)	2/73 (2.7)	
cN1	17/75 (22.7)	19/73 (26.0)	
cN2	32/75 (42.7)	33/73 (45.2)	
cN3	19/75 (25.3)	19/73 (26.0)	
Clinical M status			0.19
cM0	67/75 (89.3)	69/73 (94.5)	
cM1	8/75 (10.7)	4/73 (5.5)	
HER2-positive status	7/75 (9.3)	8/73 (11.0)	0.05
MSI status			0.06
MSS	71/75 (94.7)	70/73 (95.9)	
MSI-H	4/75 (5.3)	3/73 (4.1)	
PD-L1 CPS			0.14
CPS <5	38/75 (50.7)	42/73 (57.5)	
CPS ≥5	37/75 (49.3)	31/73 (42.5)	
Treatment exposure			
Targeted therapy use	25/75 (33.3)	23/73 (31.5)	0.04
Targeted therapy class			0.72
None	50/75 (66.7)	50/73 (68.5)	
Anti-HER2-directed	2/75 (2.7)	15/73 (20.5)	
Anti-angiogenic	23/75 (30.7)	8/73 (11.0)	
Immune checkpoint inhibitor			0.79
Sintilimab	34/75 (45.3)	45/73 (61.6)	
Cadonilimab	40/75 (53.3)	17/73 (23.3)	
Camrelizumab	1/75 (1.3)	2/73 (2.7)	
Tislelizumab	0/75 (0.0)	3/73 (4.1)	
Serplulimab	0/75 (0.0)	6/73 (8.2)	
Preoperative treatment cycles	4 (3–4)	4 (3–4)	0.15
Treatment-to-surgery interval, weeks	15 (12–16)	13 (11–15)	0.29

Values are presented as median (IQR) or n/N (%). Absolute standardized mean differences (SMDs) were used to describe between-group balance. For categorical variables with more than two levels, generalized SMDs were calculated across the overall distribution. An absolute SMD ≥0.10 was considered indicative of potential imbalance. HER2-positive status was defined as HER2 IHC 3+ or IHC 2+ with positive *in situ* hybridization, when available.

Targeted therapy was administered in 25 patients in the FLOT group and 23 patients in the SOX group (33.3% vs. 31.5%; SMD, 0.04). However, the distribution of targeted therapy classes differed substantially between groups (generalized SMD, 0.72). Anti-angiogenic therapy was more frequently used with FLOT than with SOX (23/75 [30.7%] vs. 8/73 [11.0%]), consisting predominantly of apatinib (23 vs. 7 patients) and one case of ramucirumab in the SOX group. In contrast, anti-HER2-directed therapy was more frequent with SOX (2/75 [2.7%] vs. 15/73 [20.5%]), including trastuzumab (2 vs. 10 patients) and disitamab vedotin (0 vs. 5 patients). Thus, despite similar overall rates of targeted therapy use, the specific targeted treatment backgrounds differed between groups (**Table 1**).

The distribution of immune checkpoint inhibitors also differed substantially between groups (generalized SMD, 0.79). Cadonilimab was more frequently used in the FLOT group than in the SOX group (53.3% vs. 23.3%), whereas sintilimab was more frequently used in the SOX group (45.3% vs. 61.6%). Other immune checkpoint inhibitors were uncommon in the FLOT group but accounted for 15.1% of patients in the SOX group.

The median number of preoperative treatment cycles was 4 in both groups, whereas the treatment-to-surgery interval was longer in the FLOT group (15.0 vs. 13.0 weeks).

### 3.2 Treatment-related toxicity and treatment feasibility

Treatment-related adverse events are summarized in **Table 2**, and key efficacy, toxicity, and postoperative safety outcomes are illustrated in **Figure 1**. Any-grade adverse events were common in both groups, occurring in 74 patients in the FLOT group and 68 patients in the SOX group (98.7% vs. 93.2%, P = 0.114).

**Table 2:** Treatment-related toxicity and treatment feasibility.

Variable	FLOT (n=75)	SOX (n=73)	P
Any-grade treatment-related AE	74/75 (98.7)	68/73 (93.2)	0.114
Grade ≥3 treatment-related AE	35/75 (46.7)	24/73 (32.9)	0.096
Serious adverse event	12/75 (16.0)	7/73 (9.6)	0.327
Immune-related adverse event	20/75 (26.7)	12/73 (16.4)	0.163
AE-related dose reduction/interruption	27/75 (36.0)	16/73 (21.9)	0.071
AE-related permanent discontinuation	5/75 (6.7)	5/73 (6.8)	1.000

Values are n/N (%). AE, adverse event; SAE, serious adverse event; irAE, immune-related adverse event.

The primary endpoint, grade 3 or higher treatment-related adverse events, occurred numerically more frequently in the FLOT group than in the SOX group (35/75, 46.7% vs. 24/73, 32.9%; P = 0.096). In unadjusted analysis, FLOT showed numerically higher odds of grade 3 or higher adverse events compared with SOX (OR, 1.79; 95% CI, 0.92–3.48). After adjustment for age, ECOG performance status, targeted therapy, and cadonilimab use, this association remained statistically non-significant (adjusted OR, 1.39; 95% CI, 0.67–2.87; P = 0.375).

A *post hoc* power calculation based on the observed grade 3 or higher adverse event rates (46.7% vs. 32.9%), the actual sample sizes (75 vs. 73), and a two-sided α of 0.05 yielded an estimated power of 40.5% to detect the observed between-group difference. Thus, the study had limited statistical power for the primary endpoint, and the non-significant result should not be interpreted as evidence of equivalent toxicity between FLOT and SOX.

Serious adverse events occurred in 12 patients in the FLOT group and 7 patients in the SOX group (16.0% vs. 9.6%, P = 0.327). Immune-related adverse events were also numerically more frequent in the FLOT group (26.7% vs. 16.4%, P = 0.163). Adverse event-related dose reduction or treatment interruption occurred in 27 patients treated with FLOT and 16 patients treated with SOX (36.0% vs. 21.9%, P = 0.071). Permanent discontinuation due to adverse events was similar between groups (6.7% vs. 6.8%, P = 1.000).

Overall, FLOT-based immunochemotherapy showed a numerical trend toward greater treatment-related toxicity and more frequent treatment modification, whereas permanent treatment discontinuation remained uncommon and comparable between groups.

### 3.3 Pathological response and surgical outcomes

Pathological outcomes are shown in **Table 3**. All patients underwent D2 lymphadenectomy. The median number of retrieved lymph nodes was similar between the FLOT and SOX groups (24.0 [IQR, 18.5–30.0] vs. 23.0 [IQR, 16.0–28.0]; P = 0.145), and the distribution of gastrectomy type was also comparable. Major pathological response was achieved in 32 patients in the FLOT group and 33 patients in the SOX group (42.7% vs. 45.2%, P = 0.869). Pathological complete response was observed in 14 patients treated with FLOT and 20 patients treated with SOX (18.7% vs. 27.4%, P = 0.243). The Becker tumor regression grade distribution was also similar between groups (P = 0.884).

**Table 3:** Surgical outcomes, pathological response, and postoperative safety .

Variable	FLOT (n=75)	SOX (n=73)	P
Surgical outcomes			
Type of gastrectomy			0.578
Total gastrectomy	41/75 (54.7)	34/73 (46.6)	
Distal gastrectomy	24/75 (32.0)	26/73 (35.6)	
Proximal gastrectomy	10/75 (13.3)	13/73 (17.8)	
Retrieved lymph nodes, n	24.0 (18.5–30.0)	23.0 (16.0–28.0)	0.145
R0 resection	74/75 (98.7)	73/73 (100.0)	1.000
Pathological response			
MPR	32/75 (42.7)	33/73 (45.2)	0.869
pCR	14/75 (18.7)	20/73 (27.4)	0.243
Becker TRG			0.884
TRG 1	32/75 (42.7)	33/73 (45.2)	
TRG 2	19/75 (25.3)	16/73 (21.9)	
TRG 3	24/75 (32.0)	24/73 (32.9)	
ypT stage			0.033
ypT0	14/75 (18.7)	21/73 (28.8)	
ypT1	17/75 (22.7)	7/73 (9.6)	
ypT2	6/75 (8.0)	15/73 (20.5)	
ypT3	25/75 (33.3)	21/73 (28.8)	
ypT4	13/75 (17.3)	9/73 (12.3)	
ypN stage			0.591
ypN0	46/75 (61.3)	41/73 (56.2)	0.617
ypN1	10/75 (13.3)	16/73 (21.9)	
ypN2	8/75 (10.7)	7/73 (9.6)	
ypN3	11/75 (14.7)	9/73 (12.3)	
Clinical-to-pathological downstaging	60/75 (80.0)	59/73 (80.8)	1.000
Postoperative safety			
Overall postoperative complications	16/75 (21.3)	17/73 (23.3)	0.845
Anastomotic leakage	3/75 (4.0)	3/73 (4.1)	1.000
Pulmonary infection	10/75 (13.3)	7/73 (9.6)	0.608
Abdominal effusion	10/75 (13.3)	7/73 (9.6)	0.608
Pleural effusion	6/75 (8.0)	4/73 (5.5)	0.745
Wound infection	5/75 (6.7)	6/73 (8.2)	0.763
Postoperative bleeding	5/75 (6.7)	3/73 (4.1)	0.719
Abdominal distension	14/75 (18.7)	11/73 (15.1)	0.662
Other complications	3/75 (4.0)	2/73 (2.7)	1.000
Postoperative hospital stay, days	12 (10–14)	15 (13–17)	<0.001

Values are median (IQR) or n/N (%). MPR included patients with pCR. Postoperative complications were evaluated during the index hospitalization and within 30 days after surgery. Overall postoperative complications were defined as the number of patients with at least one postoperative complication. Specific complications were not mutually exclusive.

Although the overall ypT-stage distribution differed between groups (P = 0.033), the pattern did not show a uniform shift toward lower ypT categories with either regimen. The SOX group had higher proportions of ypT0 and ypT2 disease, whereas ypT1 disease was more frequent in the FLOT group. In contrast, MPR, pCR, Becker TRG, ypN0, clinical-to-pathological downstaging, and R0 resection did not differ significantly between groups. Accordingly, the isolated difference in the overall ypT distribution was interpreted cautiously.

In unadjusted analysis, FLOT was not associated with higher odds of major pathological response compared with SOX (OR, 0.90; 95% CI, 0.47–1.73). After adjustment for age, clinical T stage, clinical N stage, clinical M status, PD-L1 combined positive score status, targeted therapy, and cadonilimab use, FLOT remained not significantly associated with major pathological response (adjusted OR, 0.68; 95% CI, 0.32–1.47; P = 0.329; **Table 4**).

**Table 4:** Logistic regression models for grade ≥3 AE, MPR, and postoperative complications.

Outcome	Event rate in FLOT	Event rate in SOX	Univariable OR (95% CI)	P	Adjusted OR (95% CI)	P	Adjusted covariates
Grade ≥3 AE	35/75 (46.7)	24/73 (32.9)	1.79 (0.92–3.48)	0.088	1.39 (0.67–2.87)	0.375	Age, ECOG performance status, targeted therapy, cadonilimab use
MPR	32/75 (42.7)	33/73 (45.2)	0.90 (0.47–1.73)	0.756	0.68 (0.32–1.47)	0.329	Age, clinical T stage, clinical N stage, clinical M status, PD-L1 CPS status, targeted therapy, cadonilimab use
Postoperative complications	16/75 (21.3)	17/73 (23.3)	0.89 (0.41–1.94)	0.775	0.80 (0.35–1.82)	0.600	Age, ECOG performance status, clinical T stage, clinical M status, targeted therapy

SOX was used as the reference group. Odds ratios greater than 1 indicate higher odds of the outcome in the FLOT group. Adjusted covariates were selected based on clinical relevance, baseline imbalance, and the number of outcome events to avoid model overfitting. P values in logistic regression models were derived from Wald tests. AE, adverse event; CI, confidence interval; ECOG, Eastern Cooperative Oncology Group; MPR, major pathological response; OR, odds ratio; PD-L1 CPS, programmed death-ligand 1 combined positive score.

These findings indicate that FLOT did not demonstrate a clear pathological efficacy advantage over SOX in this immunochemotherapy-treated cohort.

### 3.4 Postoperative safety

Postoperative safety outcomes are summarized in **Table 3**. The overall postoperative complication rate was similar between the FLOT and SOX groups (16/75, 21.3% vs. 17/73, 23.3%; P = 0.845). Specific postoperative complications were also comparable. Anastomotic leakage occurred in 3 patients in each group (4.0% vs. 4.1%, P = 1.000). Pulmonary infection occurred in 10 patients in the FLOT group and 7 patients in the SOX group (13.3% vs. 9.6%, P = 0.608). Abdominal effusion was reported in 10 and 7 patients, respectively (13.3% vs. 9.6%, P = 0.608), and wound infection occurred in 5 and 6 patients, respectively (6.7% vs. 8.2%, P = 0.763).

The median postoperative hospital stay was shorter in the FLOT group than in the SOX group (12.0 days vs. 15.0 days, P < 0.001). Because determinants of discharge timing were not systematically recorded and postoperative hospital stay was not subjected to multivariable adjustment, this difference was interpreted descriptively. In unadjusted analysis, FLOT was not associated with increased odds of postoperative complications compared with SOX (OR, 0.89; 95% CI, 0.41–1.94). After adjustment for age, ECOG performance status, clinical T stage, clinical M status, and targeted therapy, FLOT remained not significantly associated with postoperative complications (adjusted OR, 0.80; 95% CI, 0.35–1.82; P = 0.600; **Table 4**).

Thus, despite numerically higher treatment-related toxicity, FLOT-based immunochemotherapy did not appear to translate into a higher rate of postoperative morbidity.

### 3.5 Sensitivity analyses

Sensitivity analyses supported the robustness of the main findings (**Supplementary Table S1**). After excluding patients who received targeted therapy, 100 patients remained in the immunochemotherapy-only cohort, including 50 patients in each group. In this subgroup, major pathological response remained similar between FLOT and SOX (36.0% vs. 38.0%, P = 1.000), as did pathological complete response (18.0% vs. 22.0%, P = 0.803). Grade 3 or higher adverse events were numerically more frequent with FLOT (46.0% vs. 30.0%, P = 0.149), as were adverse event-related dose reduction or treatment interruption (36.0% vs. 20.0%, P = 0.118). The overall postoperative complication rate remained comparable between groups (22.0% vs. 24.0%, P = 1.000).

After excluding patients with initially metastatic disease, 136 patients with cM0 disease were analyzed, including 67 in the FLOT group and 69 in the SOX group. The two groups showed nearly identical major pathological response rates (43.3% vs. 43.5%, P = 1.000). Pathological complete response was observed in 17.9% of patients in the FLOT group and 27.5% in the SOX group (P = 0.222). Grade 3 or higher adverse events remained numerically more frequent in the FLOT group (46.3% vs. 34.8%, P = 0.221), as did adverse event-related dose reduction or treatment interruption (35.8% vs. 23.2%, P = 0.133). The overall postoperative complication rate remained similar between groups (17.9% vs. 24.6%, P = 0.405).

These sensitivity analyses showed consistent patterns: FLOT and SOX achieved similar pathological response and postoperative complication rates, whereas FLOT showed a persistent numerical trend toward greater treatment-related toxicity and treatment modification.

Sensitivity analyses stratified by individual ICI agent yielded results consistent with the primary analyses. The Mantel–Haenszel common OR for FLOT versus SOX was 1.65 (95% CI, 0.81–3.37; P = 0.165) for grade 3 or higher adverse events, 0.91 (95% CI, 0.45–1.83; P = 0.796) for MPR, 0.58 (95% CI, 0.25–1.35; P = 0.215) for pCR, and 1.04 (95% CI, 0.46–2.35; P = 0.932) for postoperative complications. Tislelizumab and serplulimab were used only in the SOX group and therefore could not contribute to within-ICI treatment contrasts.

An additional common-support IPTW analysis included 139 patients receiving ICI agents represented in both chemotherapy-backbone groups (75 FLOT and 64 SOX). After weighting, all included covariates achieved good balance (absolute standardized mean differences <0.07). IPTW-weighted ORs for FLOT versus SOX were 1.46 (95% CI, 0.74–2.92; P = 0.278) for grade 3 or higher adverse events, 0.71 (95% CI, 0.36–1.40; P = 0.322) for MPR, 0.53 (95% CI, 0.23–1.22; P = 0.138) for pCR, and 1.11 (95% CI, 0.49–2.48; P = 0.807) for postoperative complications. These weighted estimates did not materially alter the overall interpretation of the main findings (**Supplementary Table S5**).

### 3.6 Subgroup analyses

Exploratory subgroup analyses were performed according to age, PD-L1 combined positive score status, targeted therapy use, and immunotherapy background (**Figure 3**, **Supplementary Tables S2**, **S3**). Because subgroup sample sizes were small and no adjustment for multiple comparisons was performed, all subgroup analyses were considered descriptive and hypothesis-generating only. These analyses were not intended to provide confirmatory evidence of differential treatment effects across subgroups.

**Figure 3:**
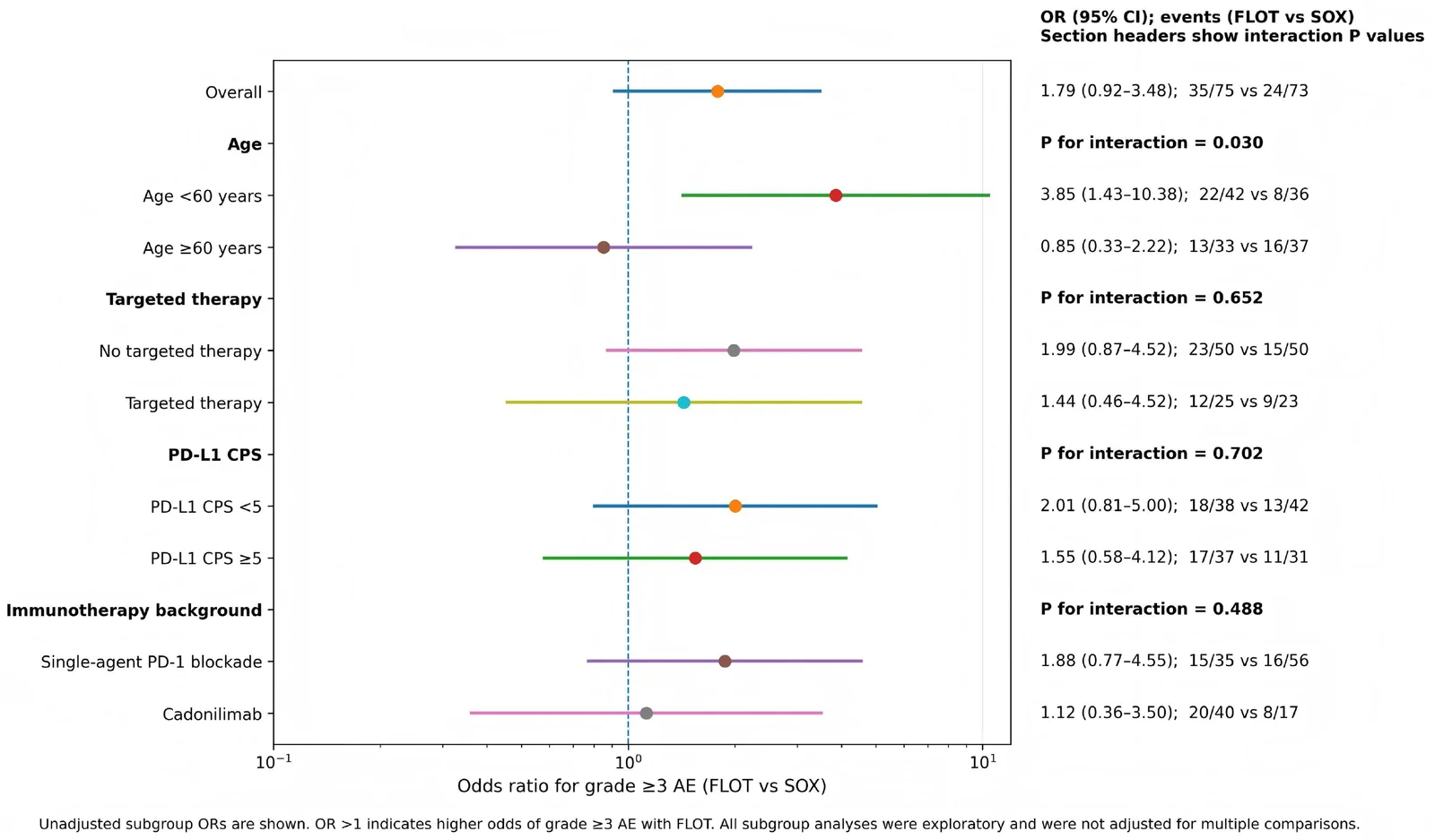
Exploratory subgroup analysis of grade 3 or higher treatment-related adverse events. Forest plot showing unadjusted odds ratios with 95% confidence intervals and event counts for grade 3 or higher treatment-related adverse events with FLOT versus SOX across clinically relevant subgroups. P values for interaction were derived from logistic regression models including treatment-by-subgroup interaction terms. All subgroup and interaction analyses were exploratory, were not adjusted for multiple comparisons, and should be considered hypothesis-generating only. AE, adverse event; CI, confidence interval; CPS, combined positive score; FLOT, fluorouracil, leucovorin, oxaliplatin, and docetaxel; OR, odds ratio; PD-L1, programmed death-ligand 1; SOX, S-1 plus oxaliplatin.

For the primary endpoint of grade 3 or higher treatment-related adverse events, unadjusted odds ratios with 95% confidence intervals across clinically relevant subgroups are shown in **Figure 3**. A nominal treatment-by-age interaction was observed (P for interaction = 0.030), with the relative odds of grade 3 or higher adverse events showing different numerical patterns between the <60-year and ≥60-year strata. However, because this interaction analysis was exploratory and was not adjusted for multiple comparisons, it should not be interpreted as confirmatory evidence of age-dependent treatment effects. No evidence of treatment-by-subgroup interaction was observed according to targeted therapy use (P for interaction = 0.652), PD-L1 CPS status (P for interaction = 0.702), or immunotherapy background (P for interaction = 0.488).

Detailed subgroup-specific rates of major pathological response, pathological complete response, serious adverse events, immune-related adverse events, adverse event-related treatment modification, and postoperative complications are provided in **Supplementary Tables S2**, **S3**. Numerical differences in these secondary outcomes were considered descriptive only, and formal interaction testing was not performed because of the limited subgroup sample sizes and the risk of multiplicity. Immunotherapy-background analyses compared FLOT and SOX within relevant treatment backgrounds and were not intended to compare different immune checkpoint inhibitors against each other.

### 3.7 Exploratory survival outcomes

Survival outcomes were analyzed as exploratory endpoints. DFS events occurred in 17 of 75 patients in the FLOT group and 15 of 73 patients in the SOX group, while 8 deaths occurred in each group. Median DFS follow-up was 23.4 months (IQR, 15.0–27.7) in the FLOT group and 8.4 months (IQR, 4.7–14.2) in the SOX group. Median OS follow-up was 24.4 months (IQR, 19.2–28.2) and 9.2 months (IQR, 4.9–15.5), respectively, demonstrating substantially lower follow-up maturity in the SOX group.

Kaplan–Meier curves with numbers at risk are shown in **Figure 4**. The log-rank P values were 0.191 for DFS and 0.357 for OS. The estimated 12-month DFS probabilities were 90.6% (95% CI, 81.3%–95.4%) with FLOT and 74.3% (95% CI, 59.7%–84.3%) with SOX. Corresponding 12-month OS probabilities were 94.7% (95% CI, 86.4%–98.0%) and 84.5% (95% CI, 70.5%–92.2%), respectively. Median DFS and OS were not reached in either group. Because of the unequal follow-up maturity, these fixed-time estimates were considered descriptive.

**Figure 4:**
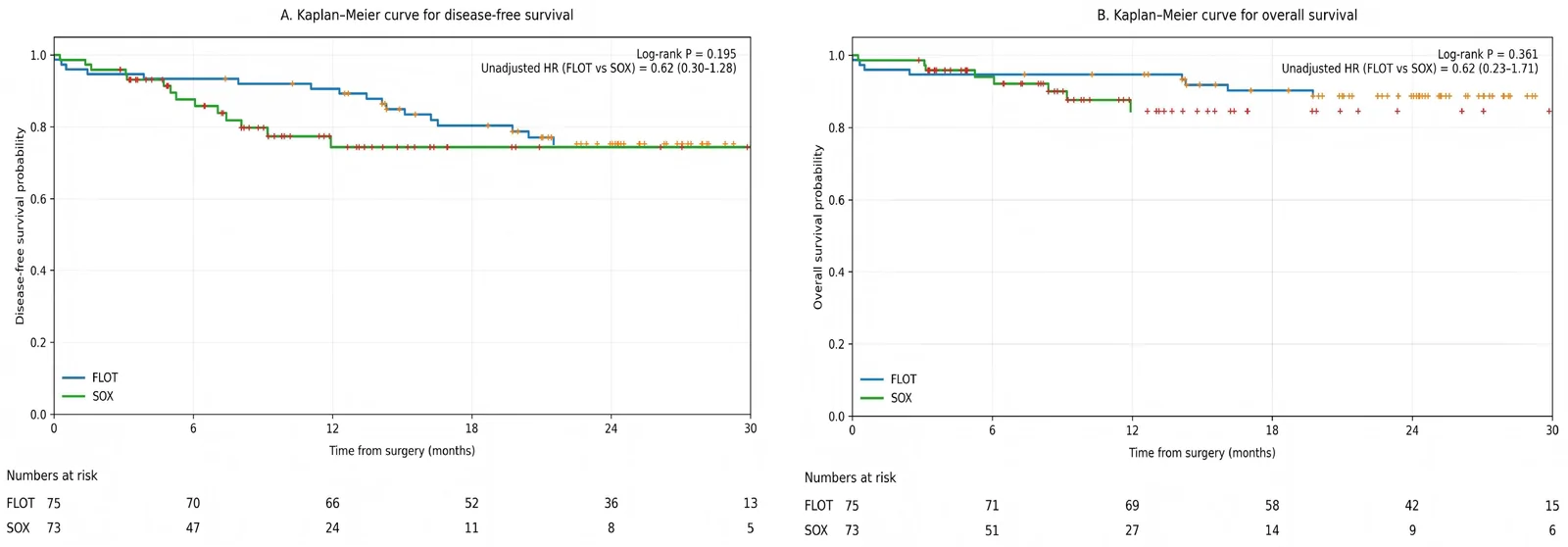
Kaplan–Meier curves for disease-free survival and overall survival. **(A)** Kaplan–Meier curve for disease-free survival in the FLOT and SOX groups. **(B)** Kaplan–Meier curve for overall survival in the FLOT and SOX groups. Tick marks indicate censored observations, and numbers at risk are shown at prespecified time points below each curve. Between-group comparisons were performed using the log-rank test, and unadjusted hazard ratios with 95% confidence intervals were estimated using Cox proportional hazards regression. Because of the limited number of events and markedly imbalanced follow-up duration between groups, particularly the shorter follow-up in the SOX group, these survival analyses should be interpreted as exploratory and should not be considered evidence of survival equivalence. Abbreviations: CI, confidence interval; DFS, disease-free survival; FLOT, fluorouracil, leucovorin, oxaliplatin, and docetaxel; HR, hazard ratio; OS, overall survival; SOX, S-1 plus oxaliplatin.

In unadjusted Cox analysis, the HR for FLOT versus SOX was 0.62 (95% CI, 0.30–1.28; P = 0.195) for DFS and 0.62 (95% CI, 0.23–1.71; P = 0.361) for OS. However, the estimates changed substantially after multivariable adjustment (**Table 5**). In the baseline-adjusted Firth Cox model, the adjusted HR was 1.09 (95% CI, 0.51–2.34; P = 0.825) for DFS and 1.50 (95% CI, 0.51–4.38; P = 0.463) for OS. After additionally adjusting for treatment-to-surgery interval, the corresponding adjusted HRs were 1.08 (95% CI, 0.50–2.34; P = 0.841) and 1.56 (95% CI, 0.53–4.60; P = 0.417), respectively. Full covariate estimates are provided in **Supplementary Table S4**. Schoenfeld residual-based diagnostics showed no evidence of non-proportionality for the chemotherapy-backbone term for either DFS (P = 0.741) or OS (P = 0.435).

**Table 5:** Cox regression analyses for disease-free and overall survival.

Outcome	Model	HR for FLOT vs SOX (95% CI)	P
DFS	Unadjusted	0.62 (0.30–1.28)	0.195
DFS	Baseline-adjusted Firth Cox	1.09 (0.51–2.34)	0.825
DFS	Extended adjusted Firth Cox	1.08 (0.50–2.34)	0.841
OS	Unadjusted	0.62 (0.23–1.71)	0.361
OS	Baseline-adjusted Firth Cox	1.50 (0.51–4.38)	0.463
OS	Extended adjusted Firth Cox	1.56 (0.53–4.60)	0.417

SOX was used as the reference group. Baseline-adjusted models included age, ECOG performance status, pretreatment cT, cN, and cM components, PD-L1 CPS status, immunotherapy background (cadonilimab vs. single-agent PD-1 blockade), and targeted therapy. Extended models additionally included treatment-to-surgery interval. Because of the limited number of survival events and sparse event distribution across immunotherapy backgrounds, multivariable estimates were obtained using Firth-penalized Cox proportional hazards regression. CI, confidence interval; DFS, disease-free survival; ECOG, Eastern Cooperative Oncology Group; HR, hazard ratio; OS, overall survival; PD-L1 CPS, programmed death-ligand 1 combined positive score.

Exploratory subgroup survival analyses are shown in **Figure 5**. Subgroup HRs varied across age, targeted therapy, PD-L1 CPS, immunotherapy background, and cM0 strata, but confidence intervals were generally wide because of the small number of events. In the cadonilimab-treated subgroup, an OS HR could not be estimated because no deaths occurred in either the FLOT or SOX subgroup during the available follow-up. Because these analyses were unadjusted, involved multiple comparisons, and were based on sparse events, they should be considered hypothesis-generating only. Overall, the survival estimates remained immature and imprecise, and no definitive conclusion regarding DFS or OS superiority, inferiority, or equivalence can be drawn from the present analysis.

**Figure 5:**
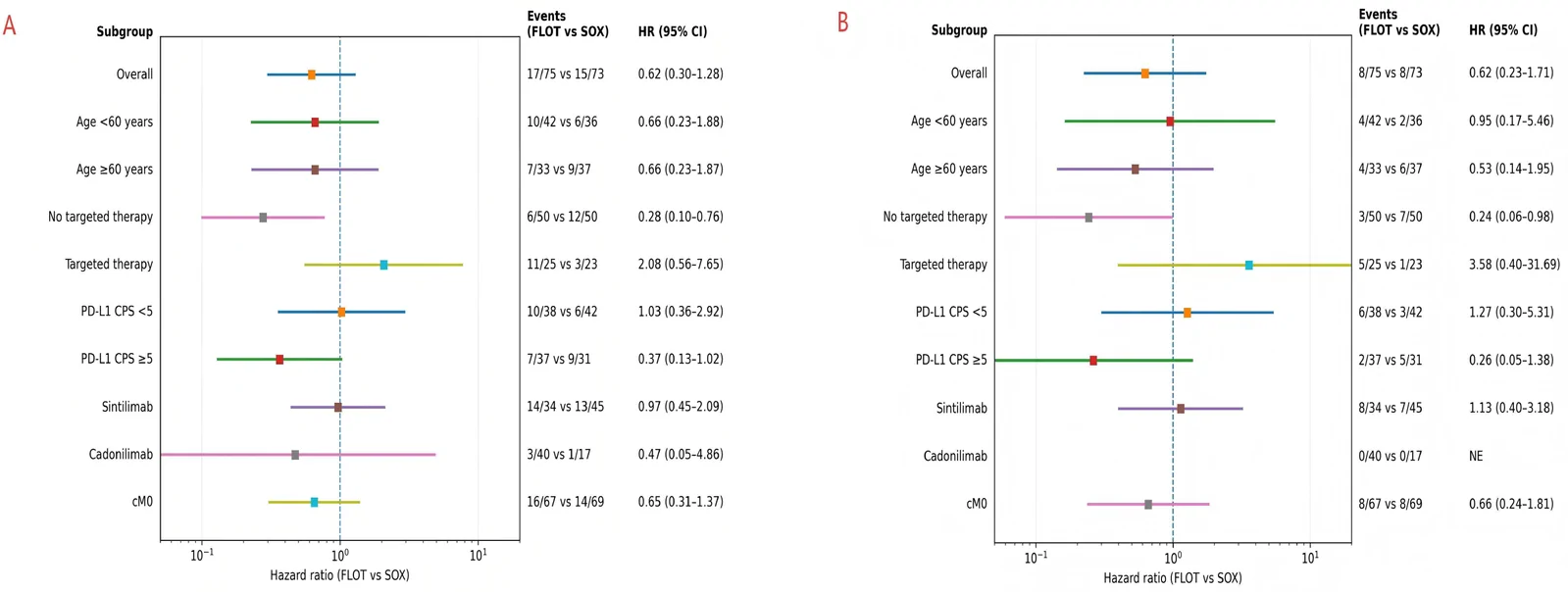
Exploratory subgroup analyses of disease-free survival and overall survival. **(A)** Forest plot showing unadjusted hazard ratios for disease-free survival with FLOT versus SOX across prespecified subgroups. **(B)** Forest plot showing unadjusted hazard ratios for overall survival with FLOT versus SOX across the same subgroups. Hazard ratios greater than 1 indicate a higher hazard of the corresponding survival event in the FLOT group, whereas hazard ratios less than 1 indicate a lower hazard. Squares represent point estimates and horizontal lines indicate 95% confidence intervals. Subgroup analyses were exploratory, were not adjusted for multiple comparisons, and were not intended to establish treatment-effect heterogeneity. Estimates based on sparse numbers of events should be interpreted with particular caution. NE indicates that the hazard ratio was not estimable because no events occurred in either treatment group within that subgroup. CI, confidence interval; CPS, combined positive score; DFS, disease-free survival; FLOT, fluorouracil, leucovorin, oxaliplatin, and docetaxel; HR, hazard ratio; NE, not estimable; OS, overall survival; PD-L1, programmed death-ligand 1; SOX, S-1 plus oxaliplatin.

## 4 Discussion

In this real-world cohort of patients with gastric cancer who received neoadjuvant or conversion immunochemotherapy followed by D2 gastrectomy, we compared FLOT and SOX as chemotherapy backbones in the immunotherapy setting. Several findings deserve attention. First, FLOT-based and SOX-based immunochemotherapy achieved broadly similar pathological efficacy, with no clear difference in MPR, pCR, ypN0, clinical-to-pathological downstaging, or R0 resection. Second, FLOT showed numerically higher rates of grade 3 or higher treatment-related adverse events and adverse event-related dose reduction or treatment interruption. Third, this greater preoperative treatment burden did not appear to translate into increased postoperative morbidity. Fourth, the apparent numerical survival trend favoring FLOT in unadjusted analyses was substantially attenuated after penalized multivariable adjustment, while sparse events and markedly imbalanced follow-up precluded definitive survival inference.

FLOT has become an important perioperative chemotherapy backbone in Western practice after the FLOT4 trial established its superiority over epirubicin-based triplet chemotherapy in locally advanced resectable gastric and gastroesophageal junction adenocarcinoma (3). This trial provided strong evidence supporting taxane-containing triplet intensification in the perioperative setting. However, the clinical context in East Asia differs substantially. D2 lymphadenectomy is routinely performed in specialized centers, and fluoropyrimidine–platinum doublets, particularly SOX, are widely used because of their tolerability, feasibility, and compatibility with D2-based surgical strategies (4, 5, 14). The RESOLVE trial supported SOX-based perioperative or postoperative treatment in patients undergoing D2 gastrectomy, further reinforcing the relevance of SOX within Asian treatment frameworks (4, 5). Therefore, whether FLOT intensification offers additional benefit over SOX in Asian D2-based practice remains a clinically relevant question.

In the present study, FLOT did not demonstrate a clear pathological efficacy advantage over SOX. MPR was achieved in 42.7% of patients in the FLOT group and 45.2% in the SOX group, while pCR occurred in 18.7% and 27.4%, respectively. Other pathological and surgical efficacy indicators, including ypN0, clinical-to-pathological downstaging, and R0 resection, were also similar between groups. The significant difference in the overall ypT-stage distribution should also be interpreted cautiously. The distribution reflected shifts across several adjacent ypT categories rather than a consistent directional reduction in pathological T stage with either regimen. Potential contributors include the modest sample size, baseline differences in clinical stage, and heterogeneity in immunotherapy and targeted-therapy backgrounds. Given the absence of consistent differences in MPR, pCR, Becker TRG, ypN status, or overall downstaging, the ypT finding alone should not be interpreted as evidence of superior pathological efficacy of either chemotherapy backbone. These findings suggest that a more intensive triplet chemotherapy backbone does not necessarily translate into superior pathological response when combined with immunotherapy. This observation is consistent with prior head-to-head evidence before the routine use of immunotherapy (10). In the Dragon III randomized phase II trial, neoadjuvant FLOT and SOX showed no statistically significant difference in pathological response or postoperative morbidity in patients with locally advanced gastric cancer (10). More recent evidence has also suggested that FLOT and SOX may yield comparable outcomes in selected patients, supporting the view that regimen selection should consider not only antitumor activity but also treatment feasibility and safety (11, 12).

The emergence of perioperative immunotherapy has further complicated the choice of chemotherapy backbone. In the final KEYNOTE-585 analysis, pembrolizumab plus perioperative chemotherapy improved pathological response and produced a favorable EFS estimate (HR, 0.81), but the EFS and OS endpoints did not meet the prespecified statistical criteria for significance (7). By contrast, MATTERHORN demonstrated a significant EFS improvement with durvalumab plus FLOT, with 24-month EFS rates of 67% versus 59% and an HR of 0.71 (9). Recent evidence therefore supports perioperative chemoimmunotherapy as an evolving treatment strategy, while also underscoring that the magnitude of benefit may depend on chemotherapy backbone, immunotherapy agent, tumor biology, and patient selection. Importantly, these trials did not directly determine whether FLOT or SOX is the preferable chemotherapy backbone for immunotherapy in Asian D2-based practice.

Our data suggest that the marginal pathological benefit of FLOT intensification may be limited in an unselected real-world immunotherapy-treated population. Both groups received immune checkpoint inhibitor-based treatment, and approximately one-third of patients in each group also received targeted therapy. Under these conditions, treatment response may be influenced by multiple components, including platinum–fluoropyrimidine chemotherapy, immune activation, tumor biology, biomarker status, disease burden, and targeted agents in selected patients. The additional contribution of docetaxel-based intensification may therefore be less apparent than expected. This does not imply that FLOT is ineffective; rather, it suggests that FLOT may not offer a universal pathological efficacy advantage over SOX when immunotherapy is already part of the preoperative regimen.

The toxicity findings are central to the interpretation of this study. Although the difference did not reach conventional statistical significance, grade 3 or higher adverse events occurred more frequently with FLOT than with SOX, and adverse event-related dose reduction or treatment interruption showed a similar numerical increase. Given the modest sample size and the *post hoc* power of 40.5% for the primary endpoint, the absence of statistical significance does not exclude a potentially clinically meaningful difference in treatment-related toxicity. This pattern is biologically and clinically plausible given the greater cytotoxic intensity of FLOT, which adds docetaxel to fluoropyrimidine and oxaliplatin (3). However, the numerical difference in treatment-related toxicity cannot be attributed to the chemotherapy backbone alone because the immunotherapy background differed substantially between groups. In routine practice, increased treatment intensity may lead to more hematologic and non-hematologic toxicity, more frequent dose adjustment, treatment delay, or temporary interruption. Therefore, when pathological efficacy appears similar, treatment-related toxicity and treatment feasibility become particularly important considerations in selecting the chemotherapy backbone.

The longer treatment-to-surgery interval observed with FLOT should also be interpreted in the context of real-world treatment decision-making. Although the median number of preoperative treatment cycles was identical between groups, the interval from treatment initiation to surgery was longer with FLOT than with SOX (15 vs. 13 weeks), occurring alongside a higher numerical rate of adverse event-related dose reduction or treatment interruption (36.0% vs. 21.9%). Toxicity-related treatment modification and the time required for recovery may therefore have contributed to the longer treatment course. However, this interval was measured from treatment initiation rather than from the final preoperative treatment cycle and thus reflects the entire preoperative treatment pathway rather than a specific toxicity-related surgical delay. In this real-world cohort, regimen selection, treatment continuation or modification, and surgical timing were individualized according to expected treatment tolerance, disease extent and response, patient preference, drug accessibility, surgical feasibility, and multidisciplinary clinical judgment. Given the greater treatment intensity of FLOT, its use may also have involved more cautious patient selection and individualized management. Because reasons for individual treatment or surgical delays were not systematically recorded, the relative contribution of adverse events versus these other clinical and practical factors cannot be determined. Accordingly, the longer treatment-to-surgery interval should be regarded as a descriptive marker of overall preoperative treatment burden rather than evidence that adverse events were the primary cause of delayed surgery.

Importantly, the greater treatment burden observed with FLOT did not translate into a higher rate of postoperative complications. Overall postoperative complications and specific complications, including anastomotic leakage, pulmonary infection, abdominal effusion, and wound infection, were similar between groups. This finding is consistent with previous evidence suggesting that neoadjuvant chemotherapy does not necessarily increase postoperative morbidity when patients are properly selected and surgery is performed in experienced centers (10). Several factors may explain this observation. First, all patients underwent surgery only after multidisciplinary reassessment, which likely excluded patients who had not adequately recovered from preoperative therapy or were not fit for gastrectomy. Second, treatment modification and adjustment of surgical timing may have allowed treatment-related toxicity to resolve before surgery. Third, all patients underwent D2 lymphadenectomy in a specialized surgical setting, consistent with established East Asian surgical standards (14). This standardized surgical approach may have reduced heterogeneity in postoperative outcomes. These findings suggest that treatment-related toxicity and postoperative morbidity should be regarded as related but distinct dimensions of safety.

The shorter postoperative hospital stay observed in the FLOT group (12 vs. 15 days) was unexpected and should be interpreted cautiously. Major surgical approach, as reflected by the distribution of total, distal, and proximal gastrectomy, was similar between groups, and lymph-node yield was also comparable, making a simple explanation based on recorded surgical extent less likely. However, baseline patient fitness differed to some extent, including an imbalance in ECOG performance status, and postoperative length of stay may also have been influenced by unmeasured factors such as recovery characteristics, discharge criteria, social or logistical considerations, and temporal changes in institutional practice during the study period. Because reasons for prolonged hospitalization and temporal changes in discharge practice were not systematically captured, the present study cannot determine the cause of this difference.

The age-stratified analyses remained exploratory. For the primary endpoint of grade 3 or higher treatment-related adverse events, a nominal treatment-by-age interaction was observed (P for interaction = 0.030). However, each age stratum contained relatively few patients, the interaction test was not adjusted for multiple comparisons, and treatment selection in this retrospective cohort was influenced by clinical fitness and physician judgment. Therefore, this finding should be regarded as hypothesis-generating rather than confirmatory evidence that age modifies the relative toxicity of FLOT versus SOX. Chronological age alone should not be used to select the chemotherapy backbone; regimen selection should instead incorporate overall fitness, ECOG performance status, comorbidities, organ function, disease burden, treatment goals, and patient preference (17).

The PD-L1 subgroup analyses were also hypothesis-generating. For grade 3 or higher treatment-related adverse events, no evidence of a treatment-by-PD-L1 CPS interaction was observed (P for interaction = 0.702). Numerical differences in pathological response and other treatment-related safety outcomes were observed across PD-L1 strata, but these secondary subgroup findings were descriptive and were not subjected to formal effect-modification testing. However, these analyses involved small numbers of patients, multiple outcomes were examined, and no adjustment for multiple comparisons was performed. Consequently, the nominal P values and numerical differences should not be interpreted as evidence that PD-L1 CPS predicts differential benefit or toxicity from FLOT versus SOX. At most, these observations provide a rationale for evaluating potential treatment-by-biomarker interactions in adequately powered prospective studies. Future adequately powered studies should determine whether PD-L1 or other biomarkers modify the relative effects of different chemotherapy backbones in the immunotherapy setting.

The imbalance in immunotherapy background represents an important potential confounder when interpreting both pathological efficacy and treatment-related toxicity. Cadonilimab was substantially more common in the FLOT group than in the SOX group (53.3% vs. 23.3%), whereas sintilimab was more frequently used in the SOX group (61.6% vs. 45.3%). Because cadonilimab provides bispecific PD-1/CTLA-4 blockade, whereas sintilimab is a PD-1 inhibitor, these immunotherapy backgrounds should not be assumed to be clinically interchangeable. In the phase III COMPASSION-15 trial in advanced gastric or gastroesophageal junction adenocarcinoma, adding cadonilimab to chemotherapy improved antitumor efficacy, while grade 3 or higher treatment-related adverse events were also common (18). Although these findings were obtained in the advanced-disease setting and do not constitute a head-to-head comparison between cadonilimab and sintilimab, they indicate that differences in immunotherapy background may influence both efficacy and toxicity estimates. Accordingly, the greater use of cadonilimab in the FLOT group may have contributed to the numerical excess of grade 3 or higher adverse events, making it inappropriate to attribute this trend solely to the chemotherapy backbone. The asymmetric immunotherapy distribution may also have influenced pathological response estimates; if dual PD-1/CTLA-4 blockade provided additional antitumor activity in some patients, its greater representation in the FLOT group could have shifted pathological response estimates in favor of FLOT, although the magnitude of such an effect cannot be determined from the present data.

Several analyses were undertaken to reduce this concern. Cadonilimab use was included as a covariate in the multivariable models for both grade 3 or higher adverse events and MPR, and FLOT remained not significantly associated with either outcome after adjustment. In addition, within the cadonilimab-treated subgroup, FLOT and SOX showed similar MPR rates (40.0% vs. 41.2%) and grade 3 or higher adverse event rates (50.0% vs. 47.1%). Within the sintilimab-treated subgroup, MPR rates were also similar (44.1% vs. 48.9%), whereas grade 3 or higher adverse events remained numerically more frequent with FLOT (41.2% vs. 26.7%). However, targeted therapy was substantially more frequent in the FLOT arm of the sintilimab subgroup, further limiting direct comparison. Together with previous real-world evidence comparing PD-1 inhibitor plus FLOT versus PD-1 inhibitor plus SOX (11), these findings suggest that the overall pattern observed in the main analysis was not obviously restricted to a single immunotherapy background. Nevertheless, these subgroup analyses were small and exploratory, and neither multivariable adjustment nor stratified analyses can eliminate residual confounding or possible interactions between chemotherapy backbone and immunotherapy agent. Therefore, the observed differences in toxicity and pathological outcomes should be interpreted as associations with the overall treatment strategy rather than isolated causal effects of the chemotherapy backbone.

The additional ICI-agent-stratified and IPTW sensitivity analyses provided further reassurance that the overall pattern was not solely driven by the unequal distribution of ICIs. Nevertheless, these methods cannot fully eliminate residual confounding, particularly because some ICI agents were used exclusively in the SOX group and because targeted therapy classes also differed substantially between treatment groups. Importantly, this study was designed to compare chemotherapy backbones rather than individual immune checkpoint inhibitors. Any apparent differences across ICI backgrounds should therefore not be interpreted as evidence of comparative efficacy or safety between specific ICI agents.

Beyond chemotherapy backbone selection, accumulating evidence also highlights the importance of biomarker-defined heterogeneity in gastric cancer immunotherapy. In advanced disease, CheckMate 649 established the clinical activity of nivolumab plus chemotherapy (19), while subsequent exploratory biomarker analyses identified associations involving molecular subtypes and stromal and immune-related gene-expression features (20). ORIENT-16 similarly demonstrated the efficacy of sintilimab plus chemotherapy, and subsequent translational analyses suggested that PD-L1 expression and features of the tumor immune microenvironment, including stromal CD8-positive immune-cell density, may further refine treatment-response assessment (21, 22). Recent meta-analyses of neoadjuvant and perioperative chemoimmunotherapy have included both FLOT- and SOX-based regimens and support the overall activity of adding immune checkpoint blockade to chemotherapy; however, regimen-level subgroup estimates from heterogeneous studies should not be interpreted as direct evidence of superiority of one chemotherapy backbone over another (23, 24). Circulating tumor DNA is another emerging biomarker. Serial perioperative ctDNA detection has shown strong associations with recurrence and survival after curative-intent treatment (25). In previously treated gastroesophageal adenocarcinoma, early on-treatment ctDNA dynamics have also shown potential for treatment-response monitoring (26). Nevertheless, ctDNA-guided therapeutic decision-making remains investigational and requires prospective validation. In the present cohort, routinely available biomarker data were primarily limited to HER2 status, MSI status, and PD-L1 CPS. Perioperative ctDNA was not routinely collected and was therefore unavailable for analysis, while other emerging molecular and immune biomarkers were also not systematically assessed. Future prospective comparisons should incorporate serial ctDNA and broader biomarker profiling to evaluate their potential roles in treatment selection and response monitoring.

The exploratory survival analyses warrant particularly cautious interpretation. In the unadjusted analyses, both DFS and OS showed HRs of 0.62 for FLOT versus SOX, which numerically suggested a lower event hazard with FLOT. However, these estimates changed substantially after adjustment for baseline clinical factors and treatment background. In the extended Firth-penalized Cox models, the adjusted HR was 1.08 (95% CI, 0.50–2.34) for DFS and 1.56 (95% CI, 0.53–4.60) for OS. This marked attenuation of the unadjusted estimates highlights the susceptibility of the crude survival comparison to confounding from nonrandom treatment allocation and heterogeneous treatment backgrounds, particularly the asymmetric distribution of immune checkpoint inhibitors. Importantly, the adjusted estimates remained highly imprecise and should not be interpreted as evidence favoring either chemotherapy backbone.

Follow-up maturity also differed substantially between groups. Median DFS follow-up was 23.4 months in the FLOT group but only 8.4 months in the SOX group, and median OS follow-up was 24.4 versus 9.2 months. Only 32 DFS events and 16 deaths were observed overall. Although the proportional hazards assumption for the chemotherapy-backbone term was not violated, statistical adjustment cannot overcome the fundamental limitations imposed by sparse events and markedly unequal observation time. Similarly, the 12-month Kaplan–Meier estimates and exploratory subgroup survival analyses are descriptive rather than confirmatory. Therefore, neither the unadjusted nor adjusted analyses provide sufficient evidence to establish superiority, inferiority, or equivalence in long-term survival between FLOT and SOX. Longer and more balanced follow-up is required before meaningful survival comparisons can be made.

This study has several strengths. First, it addresses a clinically relevant but underexplored question: the choice of chemotherapy backbone in the immunotherapy era, rather than simply whether immunotherapy should be added. Second, all patients underwent gastrectomy with D2 lymphadenectomy, reducing heterogeneity in surgical management. Third, the study evaluated multiple clinically meaningful domains, including pathological efficacy, treatment-related toxicity, treatment feasibility, postoperative safety, and exploratory survival. Fourth, sensitivity analyses excluding targeted therapy and initially metastatic disease showed consistent findings, supporting the robustness of the main results.

Several limitations should be acknowledged. First, this was a retrospective, single-center study, and treatment allocation was not randomized. The choice of FLOT or SOX was influenced by physician judgment, patient fitness, disease extent, drug availability, biomarker status, and patient preference, introducing potential selection bias. Although all patients underwent D2 lymphadenectomy and lymph-node yield was comparable between groups, detailed intraoperative measures such as operative time and blood loss were not consistently available and could not be analyzed. Second, the distribution of immune checkpoint inhibitors differed substantially between groups, with cadonilimab more frequently used in the FLOT group and sintilimab more frequently used in the SOX group. Given the different checkpoint targets of these agents, this imbalance may have confounded both pathological response and toxicity comparisons independently of the chemotherapy backbone. Although cadonilimab use was incorporated into the multivariable models for grade 3 or higher adverse events and MPR, and additional analyses were performed within the same immunotherapy background, residual confounding and potential interactions between chemotherapy backbone and immunotherapy agent cannot be excluded. Targeted therapy was also used in approximately one-third of patients, introducing additional treatment heterogeneity. Third, the exploratory subgroup analyses were based on substantially smaller sample sizes than the overall cohort. Formal treatment-by-subgroup interaction testing was restricted to the primary safety endpoint to limit multiplicity, and no adjustment for multiple comparisons was performed. Although a nominal treatment-by-age interaction was observed for grade 3 or higher adverse events, this finding may reflect chance and should be regarded as hypothesis-generating only. Secondary subgroup outcomes were therefore summarized descriptively and should not be interpreted as evidence of treatment-effect heterogeneity according to age, PD-L1 CPS, targeted therapy use, or immunotherapy background. The overall sample size was also modest, and no *a priori* sample size calculation was performed because this retrospective study included all eligible patients treated during the study period. The *post hoc* power for the primary endpoint of grade 3 or higher adverse events was 40.5% for detecting the observed between-group difference. Accordingly, the absence of statistically significant differences should not be interpreted as evidence of equivalence, particularly for outcomes with wide confidence intervals. Fourth, despite the additional penalized multivariable survival analyses, only 32 DFS events and 16 deaths were available, and follow-up maturity remained markedly imbalanced, with substantially shorter DFS and OS follow-up in the SOX group. Firth-penalized Cox regression was used to reduce small-sample bias and instability associated with sparse events, but it cannot eliminate residual confounding or compensate for limited follow-up. Survival estimates involving the immunotherapy background were particularly unstable because no deaths were observed among cadonilimab-treated patients during the available follow-up. Accordingly, both the overall and subgroup survival analyses remain exploratory and should not be interpreted as evidence of survival equivalence or treatment-effect heterogeneity. Finally, this study was conducted in a single Asian center using a D2-based surgical approach. These findings may not be directly generalizable to Western practice, where FLOT is an established perioperative standard and patterns of surgical management, including the routine implementation of D2 lymphadenectomy, may differ across centers.

Future prospective studies are needed to validate these findings and to determine whether chemotherapy backbone selection can be individualized according to patient fitness, disease burden, biomarker profile, immune microenvironment, and treatment goals. Randomized controlled trials including geographically and clinically diverse populations are required before these observational findings can be used to alter established treatment standards.

## 5 Conclusion

In this real-world cohort of patients with gastric cancer receiving neoadjuvant or conversion immunochemotherapy followed by D2 gastrectomy, FLOT and SOX showed broadly similar pathological efficacy and postoperative complication rates, while FLOT was associated with a numerically greater preoperative treatment burden. The immature and imbalanced survival data precluded definitive comparison of long-term outcomes. These findings suggest that SOX may represent a feasible immunochemotherapy backbone in selected patients within Asian D2-based practice, but they should not be interpreted as establishing superiority, equivalence, or a change in the current standard of care. Prospective randomized studies in larger and more diverse populations are required before these findings can inform changes in routine clinical practice. Exploratory subgroup findings should be regarded as hypothesis-generating only and should not be used to infer treatment-effect heterogeneity.

## Data Availability

The original contributions presented in the study are included in the article/**Supplementary Material**. Further inquiries can be directed to the corresponding author.
