# Supplementary material for: FLOT versus SOX as immunochemotherapy backbones for neoadjuvant or conversion therapy in gastric cancer: efficacy, toxicity, and postoperative safety in a real-world cohort
Source: Front Immunol. 2026 Sep 29;17:1890256. doi: 10.3389/fimmu.2026.1890256 (PMC13658598; doi:10.3389/fimmu.2026.1890256)
Supplement: Supplementary file 1 [file Table1.docx]

**Supplementary Table S1. Sensitivity analyses**

| **Subgroup / Cohort** | **Outcome** | **FLOT, n/N (%)** | **SOX, n/N (%)** | **p** |
| --- | --- | --- | --- | --- |
| Immunochemotherapy-only cohort (excluding targeted therapy) | MPR | 18/50 (36.0) | 19/50 (38.0) | 1.000 |
| Immunochemotherapy-only cohort (excluding targeted therapy) | pCR | 9/50 (18.0) | 11/50 (22.0) | 0.803 |
| Immunochemotherapy-only cohort (excluding targeted therapy) | Grade ≥3 AE | 23/50 (46.0) | 15/50 (30.0) | 0.149 |
| Immunochemotherapy-only cohort (excluding targeted therapy) | AE-related dose reduction/interruption | 18/50 (36.0) | 10/50 (20.0) | 0.118 |
| Immunochemotherapy-only cohort (excluding targeted therapy) | Postoperative complications | 11/50 (22.0) | 12/50 (24.0) | 1.000 |
| cM0 cohort (excluding cM1 disease) | MPR | 29/67 (43.3) | 30/69 (43.5) | 1.000 |
| cM0 cohort (excluding cM1 disease) | pCR | 12/67 (17.9) | 19/69 (27.5) | 0.222 |
| cM0 cohort (excluding cM1 disease) | Grade ≥3 AE | 31/67 (46.3) | 24/69 (34.8) | 0.221 |
| cM0 cohort (excluding cM1 disease) | AE-related dose reduction/interruption | 24/67 (35.8) | 16/69 (23.2) | 0.133 |
| cM0 cohort (excluding cM1 disease) | Postoperative complications | 12/67 (17.9) | 17/69 (24.6) | 0.405 |

Values are presented as n/N (%). P values were calculated using Fisher’s exact test. MPR includes patients with pCR. AE, adverse event; MPR, major pathological response; pCR, pathological complete response; cM0, clinical non-metastatic disease.

**Supplementary Table S2. Age-stratified outcomes**

| **Subgroup / Cohort** | **Outcome** | **FLOT, n/N (%)** | **SOX, n/N (%)** |
| --- | --- | --- | --- |
| Age <60 years | MPR | 18/42 (42.9) | 20/36 (55.6) |
| Age <60 years | pCR | 5/42 (11.9) | 12/36 (33.3) |
| Age <60 years | Grade ≥3 AE | 22/42 (52.4) | 8/36 (22.2) |
| Age <60 years | SAE | 9/42 (21.4) | 2/36 (5.6) |
| Age <60 years | AE-related dose reduction/interruption | 19/42 (45.2) | 7/36 (19.4) |
| Age <60 years | Postoperative complications | 13/42 (31.0) | 8/36 (22.2) |
| Age ≥60 years | MPR | 14/33 (42.4) | 13/37 (35.1) |
| Age ≥60 years | pCR | 9/33 (27.3) | 8/37 (21.6) |
| Age ≥60 years | Grade ≥3 AE | 13/33 (39.4) | 16/37 (43.2) |
| Age ≥60 years | SAE | 3/33 (9.1) | 5/37 (13.5) |
| Age ≥60 years | AE-related dose reduction/interruption | 8/33 (24.2) | 9/37 (24.3) |
| Age ≥60 years | Postoperative complications | 3/33 (9.1) | 9/37 (24.3) |

Values are presented as n/N (%). All age-stratified analyses were exploratory and hypothesis-generating. Subgroup-specific outcome rates are presented descriptively, and no within-stratum significance testing is reported because of the limited subgroup sample sizes and the absence of adjustment for multiple comparisons. Formal treatment-by-age interaction testing was restricted to the primary endpoint of grade 3 or higher adverse events and is presented in Figure 3. MPR includes patients with pCR. AE, adverse event; SAE, serious adverse event; MPR, major pathological response; pCR, pathological complete response.

**Supplementary Table S3. PD-L1 and immunotherapy-background subgroup analyses**

| **Subgroup / Cohort** | **Outcome** | **FLOT, n/N (%)** | **SOX, n/N (%)** |
| --- | --- | --- | --- |
| PD-L1  CPS <5 | Targeted therapy | 12/38 (31.6) | 14/42 (33.3) |
|  | MPR | 9/38 (23.7) | 16/42 (38.1) |
|  | pCR | 4/38 (10.5) | 11/42 (26.2) |
|  | Grade ≥3 AE | 18/38 (47.4) | 13/42 (31.0) |
|  | SAE | 8/38 (21.1) | 2/42 (4.8) |
|  | irAE | 9/38 (23.7) | 6/42 (14.3) |
|  | AE-related dose reduction/interruption | 17/38 (44.7) | 8/42 (19.0) |
|  | Postoperative complications | 10/38 (26.3) | 12/42 (28.6) |
| PD-L1  CPS ≥5 | Targeted therapy | 13/37 (35.1) | 9/31 (29.0) |
|  | MPR | 23/37 (62.2) | 17/31 (54.8) |
|  | pCR | 10/37 (27.0) | 9/31 (29.0) |
|  | Grade ≥3 AE | 17/37 (45.9) | 11/31 (35.5) |
|  | SAE | 4/37 (10.8) | 5/31 (16.1) |
|  | irAE | 11/37 (29.7) | 6/31 (19.4) |
|  | AE-related dose reduction/interruption | 10/37 (27.0) | 8/31 (25.8) |
|  | Postoperative complications | 6/37 (16.2) | 5/31 (16.1) |
| Sintilimab  background | Targeted therapy | 24/34 (70.6) | 16/45 (35.6) |
|  | MPR | 15/34 (44.1) | 22/45 (48.9) |
|  | pCR | 5/34 (14.7) | 15/45 (33.3) |
|  | Grade ≥3 AE | 14/34 (41.2) | 12/45 (26.7) |
|  | SAE | 4/34 (11.8) | 3/45 (6.7) |
|  | irAE | 7/34 (20.6) | 5/45 (11.1) |
|  | AE-related dose reduction/interruption | 11/34 (32.4) | 8/45 (17.8) |
|  | Postoperative complications | 8/34 (23.5) | 10/45 (22.2) |
| Cadonilimab  background | Targeted therapy | 0/40 (0.0) | 2/17 (11.8) |
|  | MPR | 16/40 (40.0) | 7/17 (41.2) |
|  | pCR | 8/40 (20.0) | 3/17 (17.6) |
|  | Grade ≥3 AE | 20/40 (50.0) | 8/17 (47.1) |
|  | SAE | 7/40 (17.5) | 3/17 (17.6) |
|  | irAE | 12/40 (30.0) | 5/17 (29.4) |
|  | AE-related dose reduction/interruption | 15/40 (37.5) | 6/17 (35.3) |
|  | Postoperative complications | 7/40 (17.5) | 4/17 (23.5) |

Values are presented as n/N (%). All PD-L1 and immunotherapy-background subgroup analyses were exploratory and hypothesis-generating. Subgroup-specific outcome rates are presented descriptively, and no within-stratum significance testing is reported because of the limited subgroup sample sizes and the absence of adjustment for multiple comparisons. Formal treatment-by-subgroup interaction testing was restricted to the primary endpoint of grade 3 or higher adverse events and is presented in Figure 3. MPR includes patients with pCR. The targeted therapy rows describe concomitant targeted therapy use within each subgroup. Immunotherapy-background analyses compare FLOT versus SOX within the corresponding immunotherapy background and are not intended to compare immune checkpoint inhibitors against each other. AE, adverse event; MPR, major pathological response; pCR, pathological complete response; SAE, serious adverse event; irAE, immune-related adverse event.

**Supplementary Table S4. Firth-penalized multivariable Cox regression analyses for disease-free and overall survival**

| **Covariate** | **DFS HR (95% CI)** | **P** | **OS HR (95% CI)** | **P** |
| --- | --- | --- | --- | --- |
| FLOT vs SOX | 1.08 (0.50–2.34) | 0.841 | 1.56 (0.53–4.60) | 0.417 |
| Age, per 10 years | 1.14 (0.72–1.80) | 0.580 | 1.72 (0.89–3.31) | 0.105 |
| ECOG, per 1-point increase | 3.35 (1.45–7.72) | 0.005 | 3.97 (1.26–12.47) | 0.018 |
| cT, per category increase | 1.21 (0.61–2.39) | 0.586 | 1.15 (0.45–2.93) | 0.770 |
| cN, per category increase | 1.46 (0.94–2.26) | 0.089 | 1.43 (0.80–2.54) | 0.225 |
| cM1 vs cM0 | 0.61 (0.16–2.29) | 0.467 | 0.32 (0.02–5.51) | 0.430 |
| PD-L1 CPS ≥5 vs <5 | 1.10 (0.54–2.24) | 0.788 | 1.19 (0.43–3.32) | 0.733 |
| Cadonilimab vs single-agent PD-1 blockade | 0.20 (0.07–0.62) | 0.005 | 0.03 (0.002–0.50) | 0.015 |
| Targeted therapy, yes vs no | 0.81 (0.37–1.77) | 0.596 | 0.57 (0.20–1.64) | 0.298 |
| Treatment-to-surgery interval, per week | 1.00 (0.90–1.12) | 0.929 | 0.97 (0.83–1.13) | 0.712 |

Clinical disease extent was represented by the pretreatment cT, cN, and cM components. Age was modeled per 10-year increase; ECOG performance status, cT, and cN were modeled ordinally per one-category increase; treatment-to-surgery interval was modeled per 1-week increase. The OS estimate for cadonilimab should be interpreted with particular caution because no deaths were observed among cadonilimab-treated patients during the available follow-up.

**Supplementary Table S5. Sensitivity analyses addressing immunotherapy-background imbalance**

Panel A. ICI-agent-stratified Mantel–Haenszel analyses

| **Outcome** | **ICI-stratified common OR (95% CI)** | **P** |
| --- | --- | --- |
| Grade ≥3 AE | 1.65 (0.81–3.37) | 0.165 |
| MPR | 0.91 (0.45–1.83) | 0.796 |
| pCR | 0.58 (0.25–1.35) | 0.215 |
| Postoperative complications | 1.04 (0.46–2.35) | 0.932 |

Panel B. Common-support IPTW analyses

| **Outcome** | **IPTW-weighted OR (95% CI)** | **P** |
| --- | --- | --- |
| Grade ≥3 AE | 1.46 (0.74–2.92) | 0.278 |
| MPR | 0.71 (0.36–1.40) | 0.322 |
| pCR | 0.53 (0.23–1.22) | 0.138 |
| Postoperative complications | 1.11 (0.49–2.48) | 0.807 |

Mantel–Haenszel analyses were stratified by individual ICI agent; strata without both chemotherapy backbones did not contribute to the common OR. The IPTW analysis was restricted to ICI agents represented in both FLOT and SOX groups and included 139 patients. Propensity scores included age, sex, BMI, ECOG performance status, pretreatment cT, cN, and cM status, PD-L1 CPS, targeted therapy use, and individual ICI agent. Stabilized weights were truncated at the 2.5th and 97.5th percentiles. After weighting, all absolute standardized mean differences were <0.07. CI, confidence interval; ICI, immune checkpoint inhibitor; IPTW, inverse probability of treatment weighting; MPR, major pathological response; OR, odds ratio; pCR, pathological complete response.
